# Long-term outcomes of the use of allogeneic, radiation-sterilised bone blocks in reconstruction of the atrophied alveolar ridge in the maxilla and mandible

**DOI:** 10.1007/s10561-015-9512-y

**Published:** 2015-07-11

**Authors:** Marta Krasny, Kornel Krasny, Piotr Fiedor, Małgorzata Zadurska, Artur Kamiński

**Affiliations:** Department of Orthodontics, Warsaw Medical University, Warsaw, Poland; Medicare Dental Practice, Warsaw, Poland; Department of General and Transplantation Surgery, Transplantation Institute, Warsaw Medical University, Warsaw, Poland; Department of Transplantology and Central Tissue Bank, Warsaw Medical University, Warsaw, Poland; ul. Cicha 43, 05-074 Halinów, Poland

**Keywords:** Allogeneic bone blocks, Alveolar ridge augmentation, Pre-implantation procedure, Allografts

## Abstract

Increasingly dental surgeons face the challenge of reconstruction of the height and/or thickness of the alveolar ridge as more and more patients wish to have permanent restoration of their dental defects based on intraosseous implants. Evaluation of human allogeneic bone tissue grafts in reconstruction of atrophied alveolar ridge as a pre-implantation procedure. The material comprised 21 patients aged 19–63, treated between 2009 and 2012 by the same surgeon. Restoration of bone tissue defects was performed with allogeneic, frozen, radiation-sterilised, corticocancellous blocks. The study included 26 grafting procedures with 7 procedures consisting in reconstruction of the alveolar ridge in the mandible and 19 in the maxilla. In all the cases the atrophied alveolar ridge was successfully reconstructed, which allowed placement of intraosseous implants in compliance with the initial treatment plan. After the treatment was completed the patients reported for follow-up annually. The average time of follow-up amounted to 39 months (28–50 months). None of the implants was lost during the follow-up period. There was one case of gingival recession causing aesthetics deterioration of the prosthetic restoration. In three cases the connector became unscrewed partially, which was corrected at the same visit. Frozen, radiation-sterilised, allogeneic bone blocks constitute good and durable bone-replacement material allowing effective and long-lasting reconstruction of the atrophied alveolar ridge to support durable, implant-based, prosthetic restoration.

## Introduction

Contemporary media promote people of perfect physical features. When considering facial anatomy, particular attention is paid to the smile; therefore a growing number of patients would like to have perfect dentition. In case of missing teeth restoration, when the patient reports the history of chronic inflammatory condition, traumatic extraction or congenital absence of a tooth, creating adequate ratio of the level of the gum to the length of the tooth crown as well as reconstruction of the gingival papilla may be a major challenge. The reason is reduction of the alveolar ridge dimensions, which hinders embedment of intraosseous implants (Oikarinen et al. 2003). In the described situation patients are offered safe, cheap, and quick solution—complete or partial, removable, acrylic dentures. On the other hand, in case of a borderline thickness of the alveolar process at the level of 5 mm, the embedment of an implant as well as obtaining primary and secondary stability may bring unsatisfactory aesthetic outcome.

Increasingly often a patient who was adequately informed by a doctor, does not agree to such treatment compromise and investigates chances of long-term and aesthetic, implant-based, prosthetic restoration, which depends on the amount of available bone tissue (Greenfield 1991). A place of special interest is the anterior section of the maxilla where only a slight atrophy of hard and soft tissue is acceptable (Belser et al. 2009; Kan et al. 2011). In the described situation alveolar ridge augmentation is necessary, which provides optimal ridge width and allows obtaining sufficient amount of the bone tissue from the side of the vestibule. This procedure provides a good aesthetic result of the prosthetic restoration as the treatment outcome depends not only on long-term preservation of the implant in the bone of the jaws (Pjetursson et al. 2012; Jung et al. 2008) but also on the aesthetics of the tissues surrounding the implant (Belser et al. 2009; Levin 2011).

Numerous studies proved that the patient’s own bone is the best grafting material (Misch 2011) but its use is associated with an additional surgical site which serves for harvesting the bone graft. The patient is exposed to a larger number of post-operative complications (Leonetti and Koup 2003); hence a fair number of doctors use a wide range of bone-replacement materials or allogeneic bone, which when used, eliminate the risk of complications within the donor site (Nissan et al. 2011; Leonetti and Koup 2003). The quality of the scaffold, the grade of atrophy during restructuring, and in consequence, short-term and long-term efficacy of the graft differ depending on the material used for reconstruction. Radiation-sterilised allogeneic grafts have been successfully used in reconstructive dentistry in the form of granulate (Krasny et al. 2011, 2012). However, the aesthetic outcome and efficacy of bone blocks in reconstructive dentistry have not been investigated so far.

## Objective

Evaluation of frozen, radiation-sterilised, allogeneic, corticocancellous blocks used for reconstruction of the atrophied alveolar ridge as a preparatory method before implant treatment.

## Materials and methods

The study included 21 patients aged 19–63 (42 on average), treated between 2009 and 2012 by the same surgeon. There were 15 females and 6 males in the study group. Restoration of the bone tissue defect was performed with frozen, allogeneic, corticocancellous blocks, which were radiation-sterilised with the dose of 35 kGy in the electron accelerator. Twenty-six grafting procedures were carried out, including 7 procedures consisting in the alveolar ridge augmentation in the mandible and 19 in the maxilla.

The procedures were divided into two groups. The first group comprised augmentations performed within the anterior section and the second group—procedures performed within the lateral section of the maxilla and mandible (Table 1).

**Table 1:** Distribution of augmentations depending on location

	Maxilla	Mandible	Total
Frontal section	10	2	12
Lateral section	9	5	14

### Implant preparation procedure

Preparation of bone grafts was preceded by donation, testing and procurement procedures applying to deceased donors. Donation procedure was authorised in compliance with national legislation: contact of Central Refusal Registry, contact with family to check a written statement with affixed one’s own signature, an oral statement made in the presence of at least two witnesses and confirmed by these witnesses in writing as described in article 5 of The cell, tissue and organ recovery, storage and transplantation act (2005). Donor screening was based on: reviewing of medical and social history, behavioural history, time of death, results of the autopsy and biological testing. The obligatory biological tests (Anti-HIV-1,2, HBsAg, Anti HBc, Anti-HCV-Ab and *Treponema pallidum* specific tests—TPHA) were performed in all the donors up to 24 h after death and all the results were negative. General and tissue specific donor exclusion criteria were taken into account for donor evaluation based on the tissue bank procedures adopted from EU tissue and cell directives—Directive 2004/23/EC, Directive 2006/17/EC (2004, 2006) and tissue and cell guides such as Euro-GTP and Guide to the Quality and Safety of Tissues and Cells for Human Application (2010, 2013).

Tissues including bones were procured up to 48 h after death in the local sterile field at the procurement facility of the Warsaw Forensic Department by the personnel gowned in sterile clothing using sterile surgical instruments. The procured tissues were packed in uniquely labelled double sterile foil bags and transported in transport containers at temperature of 2–8 °C with the donor’s and procurement documentation to the tissue bank. After reception in the tissue bank the procured tissues initially stored in −80 °C in deep-freezers were then released from quarantine for processing after a decision was made by a responsible person from the tissue bank following a review of the donor’s screening documentation, testing results, conditions of procurement and transportation as well as examination of tissues.

Allogeneic bone blocks were prepared in class C clean rooms in the Department of Transplantology and Central Tissue Bank. Frozen corticocancellous bone blocks were prepared from the iliac ala. Graft cleansing and shaping was carried out in clean rooms of the tissue bank at air class grade C. Bone defatting was carried out according to a validated procedure previously described (Kaminski et al. 2012). The volume of solutions used for defatting was 10 times larger than the volume of the bone. The bone samples were defatted by shaking in 96 % ethanol with 3 % of diethyl ether additive in ambient temperature for 15 min, followed by active evaporation under a hood and on absorbent paper in ambient temperature for 30 min with subsequent rinsing in saline solution at 4 °C four times, for 10 min each time. The acceptance criteria for defatting procedure is a reduction of cancellous bone weight by 75 % (±5 %), initially confirmed during validation studies of the defatting procedure effectiveness and influence of the radiation sterilisation on lipid decomposition. Validation studies were done by measuring of four hydrocarbons originating in bone marrow lipid fraction from palmitic acid (1-tetradecene and n-pentadecane) and oleic acid (8-heptadecene and 1,7-hexadecadiene) using gas chromatography with the mass spectrometry technique (GC/MS) on Agilent 7890A Gas Chromatograph system with Agilent 5975C Mass Selective Detector, Agilent 7683 B Series Injector and Agilent 7683 series Autosampler (Agilent Technologies, Inc., USA).

Bone grafts were double packed in foil (polyester/polythene) and subsequently stored frozen in −80 °C deep-freezer until sterilisation. The bone grafts were radiation-sterilized with accelerated electron beam (35 kGy) in an accelerator (LAE-10; 10 MeV) in the Institute of Nuclear Chemistry and Technology in Warsaw, Poland on dry ice in temperature of −60 °C. Validation of the sterilisation procedure was based on ISO norms (ANSI/AAMI/ISO 11137-1:2006a, ANSI/AAMI/ISO 11137-2:2006b, ANSI/AAMI/ISO 11137-3:2006c) and performed by the Central Tissue Bank in cooperation with a radiation facility. The sterilisation dose was established based on validation studies as well as taking into consideration radiation inactivation of viral pathogens (Fideler et al. 1994; Tomford et al. 1981; Pruss et al. 2002).

Transport of tissues to and from irradiation facility was performed in frozen state in the containers with dry ice keeping temperature below −60 °C. After sterilisation bone grafts were placed back in the −80 °C deep-freezer and stored there until distribution.

### Surgical procedure

**Fig. 1:**
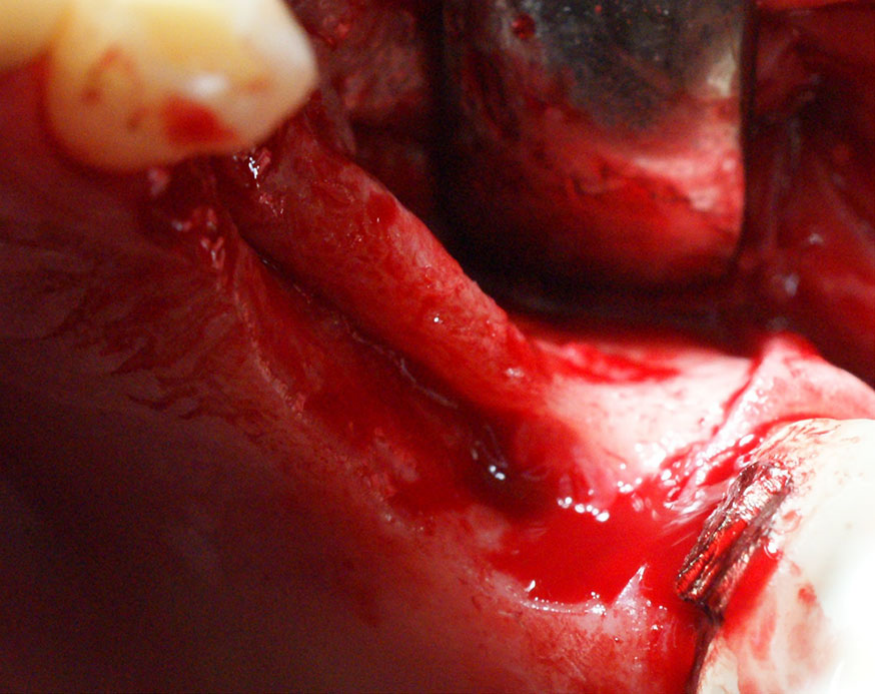
Atrophied alveolar ridge before augmentation

Under local anaesthesia with 4 % Ubistesine forte an incision of the mucous membrane was made within the area of planned bone grafting using the palatal approach in the maxilla and lingual approach in the mandible. Horizontal incisions were made going across the periodontium of the adjacent teeth and then vertically to the vestibular fornix in the maxilla or in the mandible. After the mucoperiosteal flap was detached, the recipient area was revealed (Fig. 1).

The procedures were performed with the use of allogeneic, corticocancellous blocks. Then, the bone block was processed with the use of straight handpiece burs. The graft was prepared so the surface of the cancellous bone matched the recipient site accurately and the lamina dura was located from the side of the vestibule and the alveolar ridge apex. In view of the graft resorption during its reorganisation the thickness of the block was selected so the widening was approximately 30 % greater than the optimal required transversal dimension. In order to seal the contact surface between the ridge bone and the bone block more accurately before the fixing screws were finally tightened up, ground cancellous bone composed of shavings from the processed bone block was used to fill this space.

**Fig. 2:**
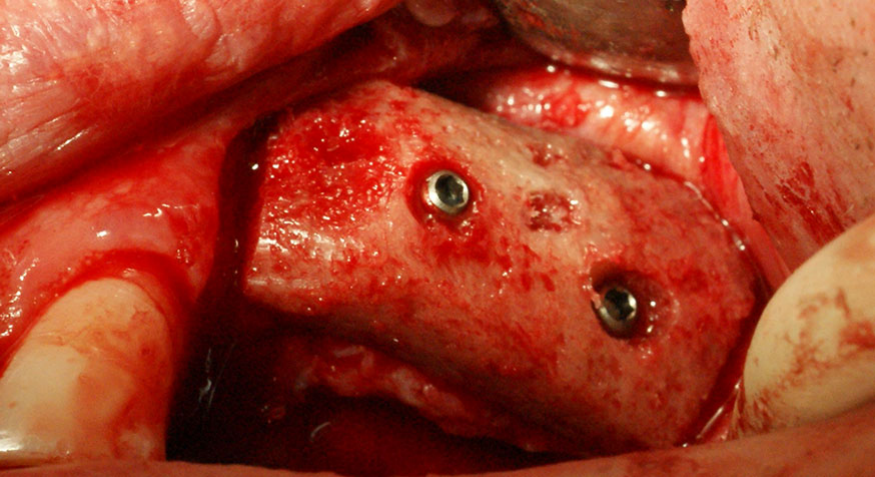
Bone block fixed to the ridge with two screws

The bone block was fixed with screws from the Meisinger Transfer Control kit (Fig. 2). The number of screws depended on the size of the block but was never smaller than 2 to prevent rotation of the block caused by pressure exerted by prosthetic restoration or morsels on this site. The patients were informed there would be a thickening in the oral vestibule corresponding to the screw head. That did not lead to perforation or inflammation of the mucous membrane within the area. Finally the sharp edges of the bone block were smoothed out with a straight handpiece bur with small notches.

The entire area was covered with PRF membranes obtained from the patient’s blood. PRF membranes were obtained from the patient’s blood drawn into vacuum tubes and then centrifuged at 2700 g for 12 min. After the supernate was discarded, PRF membranes were obtained showing relatively high tensile strength and increased viscosity (Krasny et al. 2011). Before repositioning the mucoperiosteal flap was extended by adequate longitudinal incisions of the periosteum to prevent excessive tension within the mucous membrane.

Post-operative recommendations were aimed mainly at reduction of oedema and pain complaints, reinforcement of gum healing but the most important ones referred to antibacterial protection. The patients were instructed to take Augmentin 1000 bid for 7 days.

**Fig. 3:**
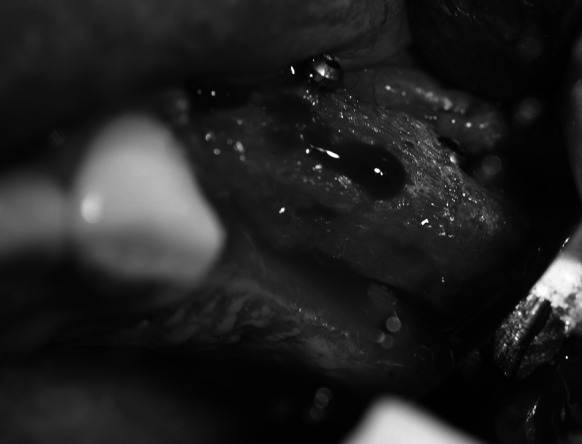
Healed bone block graft

**Fig. 4:**
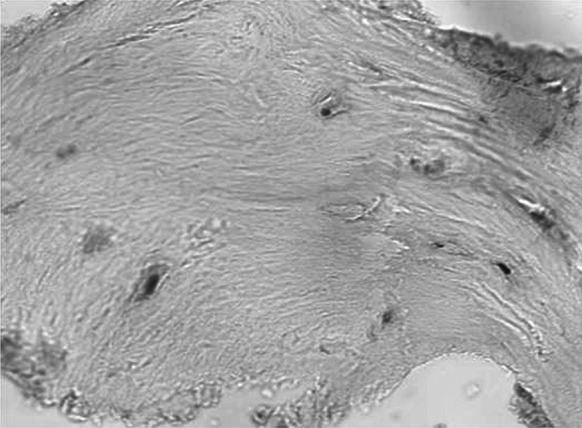
The tissue specimen from the site of implant embedment previously reconstructed with a biostatic bone graft. The graft restructuring can be confirmed by the occurrence of osteocytes in the lacunae. ×400 magnification, H&E staining

Following 3–6 moths required for graft restructuring (Fig. 3), jointly 33 implants (BIOMET 3I) were placed in the regenerated alveolar ridge. Bone shavings from the bur (remaining there after the bed for the implant was prepared) were subjected to histopathological examination. (Fig. 4). After osteointegration which took 3 months in case of the mandible and 6 months in case of the maxilla, all the implants were weighed down with prosthetic crowns.

## Results

In all the cases the primary transverse dimension of the alveolar ridge ranged from 1 to 4 mm (3 mm on average), which did not meet the minimum standard determined by the diameter of the implant plus 1.5 mm of bone tissue from the side of the vestibule and tongue/palate. The average dimension of the ridge following augmentation amounted to 8.7 mm (7.3–10 mm) (Table 2).

**Table 2:** Dimensions of the alveolar ridge in millimeters before and after augmentation

	Maxilla		Mandible	
	before	after	before	after
Frontal section	3.2	9	1.5	6.6
Lateral section	2.9	8.4	2.8	8.9

**Fig. 5:**
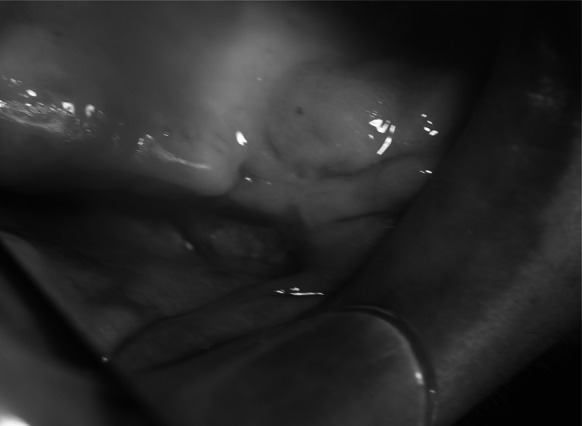
The bone block being partially exposed during the healing process

In two cases at the stage of integration of the block with the recipient site an abrasion of mucous membrane was found with hard tissues revealed (Fig. 5). A minor procedure was performed consisting in smoothing the surface of the block within this area and mucoplasty which allowed normal graft healing. In one case after 2 weeks of augmentation while the adjacent tooth was endodontically treated the bone block fixation was damaged during cofferdam installation. This case required another procedure of augmentation.

**Fig. 6:**
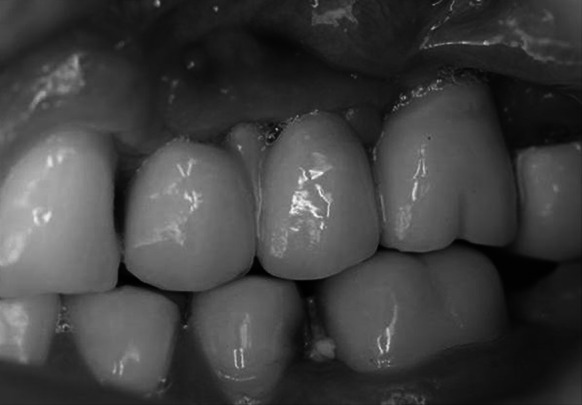
Aesthetics of prosthetic restoration 2.5 years after the treatment was completed

The follow-up period ranged from 28 to 50 months (39 months on average). At the annual follow-up visits implant stability, clinically evaluable bone atrophy, and aesthetics of the restoration were examined. The mesial papilla, distal papilla, soft tissue level, soft tissue contour and colour were investigated. A 2 years after the implant-prosthetic treatment was completed eight patients showed improved aesthetics compared to the day of restoration completion (Fig. 6), when the pressure exerted by the crown on the gum induced inadequate blood supply in the adjacent soft tissue, which deteriorated the aesthetic evaluation of the restoration. In eleven cases the aesthetics remained at the same level. In two cases a dark rim was visible around the neck of the prosthetic crown, which was regarded as slight deterioration of the aesthetics. However, all the patients declared that they were satisfied with the aesthetic outcome of the treatment.

During the follow-up period there were three cases where the connector got loosened, which was corrected at the nearest visit of the patient. The aesthetics throughout the entire follow-up period deteriorated only in one case of a 65-year-old male, who showed 1 mm gingival recession at the side of the vestibule (lateral section of the mandible). However, there were no cases of implant stability deterioration or lost implants.

## Discussion

Autogenous fresh bone grafts constitute a golden standard in alveolar ridge augmentation but the limited amount of the available material as well as the risk to benefit ratio in view of complications related to the donor site often persuade the doctor to seek alternative solutions. The use of allogeneic bone blocks for reconstruction of the atrophied alveolar ridge seems to be an effective method, the efficacy of which is comparable to the one of the autogenous material as numerous studies confirm (Schlee et al. 2014; Peleg et al. 2010; Krasny et al. 2011, 2014). Allogeneic bone blocks guarantee long-term stability of hard and soft tissues in patients who underwent allogeneic bone block grafting (Schlee et al. 2014), which was also demonstrated in this study. The density of the obtained bone tissue within the area of allo-grafting was acceptable; therefore this technique could constitute an alternative for auto-grafting (Lumetti et al. 2014). The reconstructed ridge was not resorbed if the bone graft was adequately prepared, which was confirmed by implant survival at the level of 90 % after 10 years of the procedure (Macedo et al. 2012). Data obtained in the presented study showed 100 % of preserved bone blocks 2.5 years after grafting.

Not only the long time of use but also the quality of the prosthetic restoration is important for the patient. The objective measure of aesthetics is the gingival level, proportions of crowns in adjacent teeth as well as occurrence of gingival papillas. In this study the aesthetics of restoration was maintained at a satisfying level in nearly 90 % of patients throughout the follow-up. This indicated low clinical atrophy of the reconstructed alveolar ridge, which extended the lifespan of dental implants embedded in the ridge.

Efficacy of the grafting procedure is closely related to experience of the surgeon and the technique of graft preparation (Peleg et al. 2010). Correct choice of a block has crucial implications for the degree of atrophy during reorganisation. The cancellous layer of the graft facilitates revascularisation, whereas the compact bone provides adequate resistance to forces affecting the facial skeleton. The combination of the two layers makes the graft structure less prone to atrophy during restructuring. It is also very important to prepare the surfaces of the graft as well as the recipient site, which must fit together precisely after the block is fixed. It allows more efficient restructuring.

Resorption of an allogeneic bone graft during its restructuring depends also on the method of preparation in the tissue bank. Radiation-sterilisation weakens the collagen structure which forms a scaffold for growth and differentiation factors, including BMP-2, participating in graft restructuring. The degree of this damage depends mainly on the ionizing radiation dose used for sterilisation and also on the temperature of radiation and whether the graft contains water. It seems that due to the direct effect of free radicals on tropocollagen molecules its chains are cut and this result depends on the radiation dose. The higher the dose, the more severe the damage is (Bailey 1968; Bright and Burstein 1978; Dziedzic-Goclawska et al. 2005). At the same time, if the graft contains water (i.e. was not lyophilised) in an indirect mechanism subsequent to water radiolysis, due to the effect of hydroxyl radicals, immature cross links are formed in collagen which stabilise its structure (Salehpour et al. 1995; Dziedzic-Goclawska et al. 2005). On the other hand, low temperature sterilisation causes immobilisation of the free radicals and weaker reactions with the collagen molecule. Therefore, low temperature of radiation protects the structure of graft collagen. Then again it must be remembered that low temperature sterilisation weakens the effectiveness of microorganism inactivation; thus the sterilisation dose should be higher (Pruss et al. 2002). Although there are publications describing the use of lower doses of ionising radiation for graft sterilization determined according to ISO/AAMI/ANSI 11137, the methodology of establishing the sterilizing dose took only bacteria inactivation into account. Alcohol used for the defatting procedure mentioned above, due to its concentration, is not considered an inactivating substance but a means, the sole purpose of which is to remove the marrow and adipose tissue from the graft.

The dose of 15 kGy may inactivate the HIV, but it seems that higher doses should be used (Knaepler et al. 1992). The dose of 30 kGy (Fideler et al. 1994) or even over 30 kGy (Tomford 1981) has been shown to be effective against HIV. Therefore, considering the abovementioned papers on viral inactivation, as well as the risk of viremia being in a window period with negative serological test results for viral infections during evaluation of the potential donor, the authors used the dose of 35 kGy to sterilise grafts.

In the presented material none of the implants were lost during the follow-up period and considerable deterioration of the restoration aesthetics was not observed. The restoration aesthetics was maintained at a satisfying level as in similar studies published by other authors (Gu et al. 2014). The case of gingival recession in one patient seemed to have no relation with the type of material used as this category of changes were also observed by other authors who used autogenous material for grafting (Pieri et al. 2013).

## Conclusions

Frozen, radiation-sterilised, corticocancellous blocks constitute a good, durable, and predictable bone-replacement material. An adequate technique of block preparation during augmentation as well as the use of allogeneic shavings to seal the borderline between the graft and the patient’s bone allows reduction of the extent of block mass resorption during its reorganisation. Owing to that property a desired shape of the alveolar ridge may be obtained which allows aesthetic restoration of the dental defect possible to be maintained at a satisfying level throughout a long time.
